# Biological Activity Analysis of Native and Recombinant Streptokinase Using Clot Lysis and Chromogenic Substrate Assay 

**Published:** 2012

**Authors:** Arash Mahboubi, Seyyed Kazem Sadjady, Mohammad Mirzaei Saleh Abadi, Saeed Azadi, Roya Solaimanian

**Affiliations:** a *Department of Pharmaceutics, School of Pharmacy, Shahid Beheshti University of Medical Sciences, Tehran, Iran. *; b *Pharmaceutical Sciences Research Center, Shahid Beheshti University of Medical Sciences, Tehran, Iran.*; c *Pharmaceutical Sciences Branch, Islamic Azad University, Tehran, Iran. *; d *Darou Pakhsh Pharmaceutical Manufacturing Company, Biotech Research Department. Tehran, Iran.*; e *Darou Pakhsh Pharmaceutical Manufacturing Company, Quality Control Department, Tehran, Iran. *; f *Student Research Committee, School of Pharmacy, Shahid Beheshti University of Medical Sciences, Tehran, Iran. *

**Keywords:** Native streptokinase, Recombinant streptokinase, Clot lysis, Chromogenic substrate, Biological activity

## Abstract

Determination of streptokinase activity is usually accomplished through two assay methods: a) Clot lysis, b) Chromogenic substrate assay. In this study the biological activity of two streptokinase products, namely Streptase®, which is a native product and Heberkinasa®, which is a recombinant product, was determined against the third international reference standard using the two forementioned assay methods. The results indicated that whilst the activity of Streptase® was found to be 101 ± 4% and 97 ± 5% of the label claim with Clot lysis and Chromogenic substrate assay respectively, for Heberkinasa® the potency values obtained were 42 ± 5% and 92.5 ± 2% of the label claim respectively. To shed some light on the reason for this finding, the *n-*terminal sequence of the streptokinase molecules present in the two products was determined. The results showed slight differences in the amino acid sequence of the recombinant product in comparison to the native one at the amino terminus. This finding supports those of other workers who found that *n-*terminal sequence of the streptokinase molecule can have significant effect on the activity of this protein.

## Introduction

Streptokinase (SK) was the first thrombolytic drug to be introduced as a treatment for acute myocardial infarction more than 45 years ago (1). It is now the leading fibrinolytic agent in the treatment of thromboembolic conditions and is included in the World Health Organization (WHO) Model List of Essential Medicines (2). It is cheap to produce and is manufactured in many countries around the world (3). However, its quality control, particularly determination of its biological activity, can be more complicated than generally assumed (4). Streptokinase, a 47-kDa protein produced by various strains of *β*-hemolytic streptococci, interacts with and activates human plasminogen to form an active complex capable of converting other plasminogen molecules to plasmin (5,6). 

Streptokinase consists of 440 amino acids, including a 26-amino acid *n-*terminal signal peptide which is cleaved during secretion to yield the mature 414 amino acid protein (7).

The potency of streptokinase is determined by comparing its capacity to activate plasminogen to form plasmin with the same capacity of international reference standard applying two possible methods: a) Fibrin clot lysis assay, b) Chromogenic substrate assay (without fibrin). The fibrin clot lysis method is based on clotting of fibrinogen in the presence of plasminogen and streptokinase (8). In the second assay, plasminogen activation is measured using the plasmin-specific substrate S-2251™ (9).

In this study, two different streptokinase products were evaluated. The first one, Heberkinasa®, is a recombinant streptokinase product manufactured by HEBER BIOTECH, a company in Cuba. It is obtained through the isolation and cloning of the streptokinase gene of a strain of *Streptococcus equisimilis *group C and its expression in *E. coli*. It contains five mutated amino acids in comparison to the native streptokinase (10, 11). The second product tested, Streptase®, on the other hand, is produced by CLS Behring GmbH, Germany, and is a highly purified streptokinase derived from the culture filtrate of beta-hemolytic streptococci of Lancefield group C. Due to it being a native product as well as its strong fibrinolytic activity, it has frequently been used as the reference product for comparing the potency of a number of streptokinase preparations (12).

The dose of fibrinolytic agents must be carefully controlled since too low or too high a dose may have potentially serious clinical implications (1). On the other hand in order to determine the correct dose of SK products their biological activity must be determined with reasonable accuracy. The objective of this study was, therefore, to evaluate the effect of the assay method used on the potency value of both a recombinant and a native streptokinase product.

## Experimental

*Streptokinase*

15 vials of two preparations of three batches of streptokinase (five vials for each batch), namely Streptase® (CLS Behring GmbH, Germany) and Heberkinasa® (HEBER BIOTECH, Cuba) both containing 750000 IU per vial as a lyophilized powder, were tested. The 3rd international standard of streptokinase 00/464 (WHO/NIBSC-UK EN63QG) containing 1030 IU per vial was used as reference standard.

*Clot lysis assay*

First the fibrin plate was prepared in the following manner:

Two solutions ((a) and (b)) were prepared as follows: 

a) 200 mg of agarose (Merck, Germany) was dissolved in 20 mL phosphate buffer solution with pH of 7.2 and maintained at 56°C.

b) 50 mg of human fibrinogen (Fibrinogen, Fraction 1, Type 1 from Human Plasma, Sigma-Aldrich, USA) was dissolved in 10 mL phosphate buffer solution (pH = 7.2).

Twenty hundred μL of 1 mg/mL human plasminogen (Human plasminogen, Hyphen Biomed, France) was added to solution (a) and 500 μL of thrombin (10 NIH unit/vial, from human plasma, Sigma-Aldrich, USA) to solution (b). Clot formation was initiated by mixing solutions (a) and (b) in a Petri dish. It was maintained at 4°C for 30 min. Dilutions, over the range 100-1000 IU/mL, were prepared from the international standard with Milli-Q water and loaded, as duplicate, onto fibrin plate and incubated for 8 h. The zones of lysis produced on the fibrin plates were measured and related to concentration to obtain the dose-response curve for streptokinase. The test samples were prepared by reconstitution of each vial with 1 mL of Milli-Q water. Using the dose-response curve, the streptokinase activity of the samples was determined from zones of lysis produced by appropriate dilutions of each sample loaded on fibrin plates.

*Chromogenic substrate assay*

The chromogenic method is an endpoint method using a chromogenic substrate (S-2251 (Val-leu-lys-p-nitroaniline.2HCl); Chromogenix, Milan, Italy), where Streptokinase converts plasminogen to plasmin in solution, and in the absence of fibrin. In this method, a given concentration of Streptokinase results in linear generation of active plasmin and accelerating hydrolysis of S-2251, hence resulting in the optical density (OD) of the solution increasing exponentially.

The total amount of product formed, and hence the OD, is proportional to the Streptokinase concentration. Substrate solution was prepared by adding 1 mL of 0.5 M Tris-HCl with pH of 7.4 at 37°C to 1 mL of 3 mM S-2251 (reconstituted in water) and 5 μL of 10% Tween 20 (Usb®,USA). This solution was maintained at 37°C and, immediately before use, 45 μL of plasminogen solution (1 mg/mL) were also added. Dilutions for the dose-response curve for Streptokinase were prepared in 10 mM Tris-HCl with pH of 7.4 containing 0.1 mM NaCl and 1mg/mL albumin, over the range 4.0, 2.0, 1.0, 0.5 IU/mL and maintained at 37°C in a microtiter plate. Plasminogen activation was initiated by mixing 60 μL of Streptokinase solution with 40μl of substrate solution. Concentrations of Streptokinase in the activation mixture were, therefore, 2.4, 1.2, 0.6, 0.3 IU/mL. The reaction was allowed to proceed for 20 min and then stopped by addition of 50 μL of 50% acetic acid and the OD was measured at 405 nm (9).

*N-terminal sequencing*

For *n*-terminal sequencing, native PAGE (Mini-Protean® 3 cell, Bio-Rad, USA) was carried out on 10% polyacrylamide gel followed by Western blotting (Trans-Blot®, Bio-Rad, USA) onto PVDF membrane. Then, the sequence of *n-*terminal region of the two products was analyzed by Edman degradation method using a micro sequencer (Applied Biosystems, Procise Model 492).

## Results and Discussion

**Figure 1:**
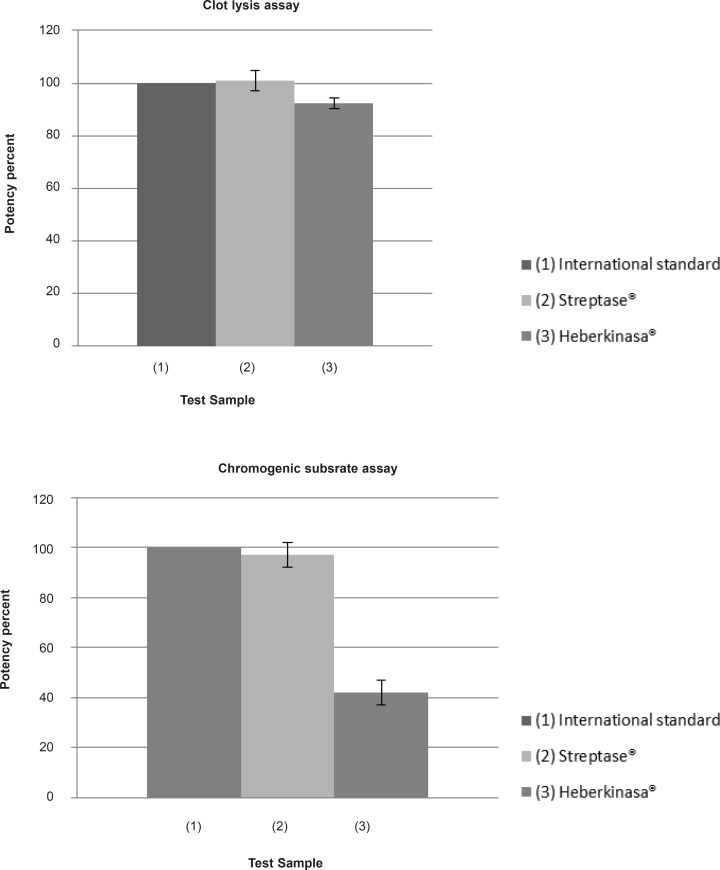
The streptokinase products activity assessed by fibrin-clot and chromogenic methods. Analysis of biological activity of native and recombinant streptokinase using Clot lysis and Chromogenic substrate assay

In the following Myocardial Infarction, if the correct dose of Streptokinase is administered within a certain time, the chances of survival increases considerably. Therefore, it is vital to be able to assign accurate potency values to Streptokinase preparations. In the present study the activity of two products (native vs. recombinant) were determined by two different methods. The average of potency results obtained for the two samples using the two methods are given in Table 1 and presented diagrammatically in Figure 1. 

**Table 1:** Potency results and *n*-terminal sequencing of two streptokinase preparations

**Sample**	**Chromogenic substrate assay**	**Clot lysis assay**	***N*** **-terminal sequence**
Streptase®	97 ± 5%	101 ± 4%	I-A-G-P-E-W
Heberkinasa®	42 ± 5%	92.5 ± 2%	M-I-A-G-P-E

The activity of Streptase® was found to be 101 ± 4% and 97 ± 5% of the label claim with Clot lysis and Chromogenic substrate assay respectively. These results are within the acceptable limits of the European pharmacopeia requirements of potency (90-111% of stated potency). For Heberkinasa® the potency values obtained were 42 ± 5% of the label claim with the Chromogenic substrate assay and 92.5 ± 2% with the Clot lysis test. However, in a recent international collaborative study, both methods gave equivalent results when the third international standard for streptokinase was being evaluated (9).

**Figure 2:**
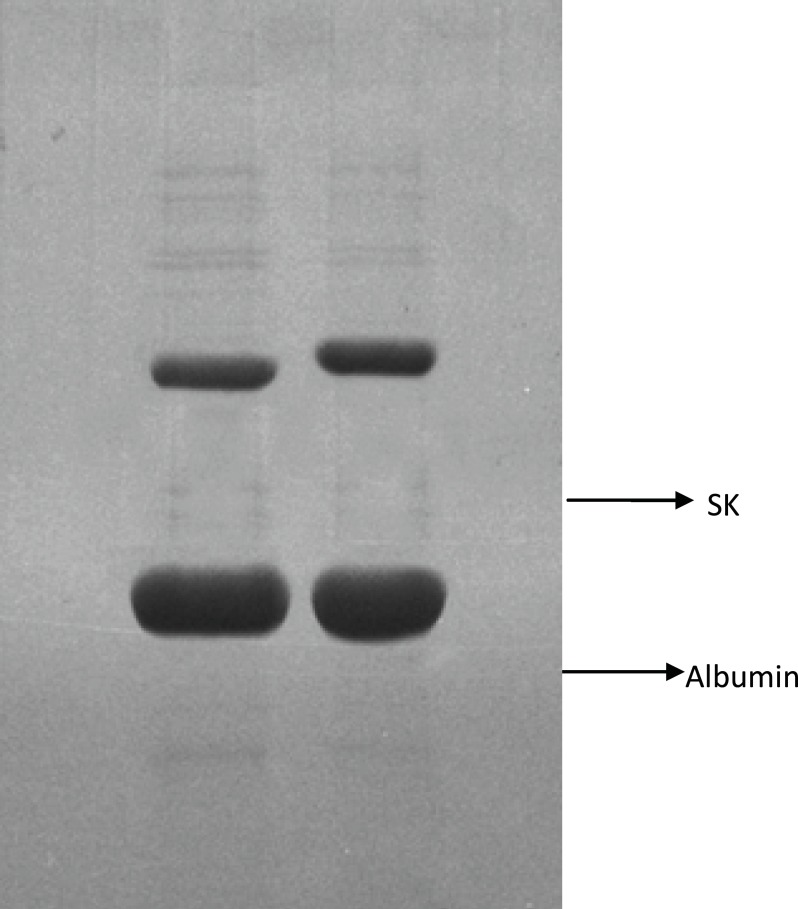
Native PAGE analysis of two streptokinase products. Lane (A) Heberkinasa® Lane (B) Streptase®.Analysis of biological activity of native and recombinant streptokinase using Clot lysis and Chromogenic substrate assay

**Figure 3:**
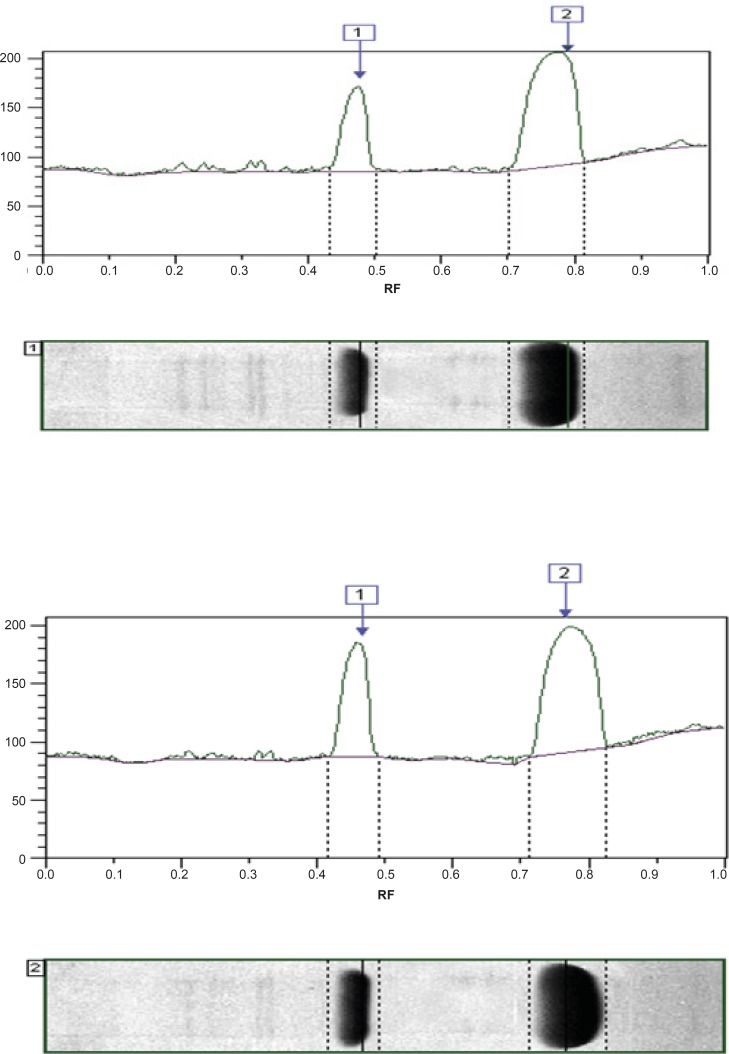
Densitometric comparison of two preparations on native gel stained with coomassie blue. (A) Heberkinasa®, (B) Streptase® Analysis of biological activity of native and recombinant streptokinase using Clot lysis and Chromogenic substrate assay

In order to investigate the reason for the different results obtained in the present work in comparison to the aforementioned study, Native-PAGE analysis of the two products was carried out (Figures 2 and 3). 

The results showed that the recombinant SK, present in Heberkinasa®, moved more rapidly on the gel than the native form contained in Streptase®. This finding suggests that the structure of the two forms of SK is, to some extent, different. N-terminal sequencing of the two proteins confirmed this finding. The sequencing of the first 6 amino acids revealed the presence of an additional methionine (Met) in the recombinant protein (Table 1).

In other studies these assay discrepancies have also been attributed to the exact sequence of the *n-*terminal amino acid residues in streptokinase which is known to be important for plasminogen binding and activation (13) by a mechanism termed ‹molecular sexuality (14). The first amino acid residue in *n-*terminus of native streptokinase is Ile. Establishment of a salt bridge between Ile1 of streptokinase and Asp740 of plasminogen is necessary for streptokinase to induce an active site in plasminogen by a non-proteolytic mechanism. In two studies mutation of Ile1of streptokinase to an alanine caused a decrease in plasminogen activity (to 23 ± 7% that of wild-type SK) (7, 14). If, as a result of incomplete processing of the protein in E.coli, Ile1 is substituted with methionine at the protein›s *n-*terminal, recombinant streptokinase may exhibit a different profile of activity and fibrin-dependency (16, 17). Other studies have also suggested that small modifications in the sequence of native streptokinase at the amino- or carboxy-terminal can have significant effects on its activity and fibrin-dependence. In the present study, the native form of streptokinase, which is contained in Streptase® and has identical sequence with international standard, exhibited similar results using both assay methods (with or without fibrin). On the other hand, the recombinant streptokinase present in Heberkinasa®, with the *n-*terminal methionine, showed discrepant results, i.e. the assay result depended on the type of test employed and possibly the presence of fibrin in the method.

In a multi-center study, the 3rd IS was calibrated using various assay systems, including chromogenic substrate and fibrin clot lysis assay 9. The results of this collaborative study showed acceptable conformity within and between the laboratories, with no considerable differences between the different methods. Based on the recommendations from the aforementioned collaborative study, the chromogenic method, which does not include fibrin, became the European Pharmacopoeia (EP) method for streptokinase potency determination from January 2004 and the British Pharmacopoeia (BP) method from December 2004, when it replaced a method involving fibrin clot (18, 19). Analysis of the results from the collaborative study to establish the 3rd international standard showed that the fibrin-dependent test (clot lysis) was not necessary where the test streptokinase and the international standard were identical, i.e. of the native type. However, where recombinant streptokinase is not identical to the international standard, because of incomplete processing or making changes to the molecule to either reduce immunogenicity or to develop new variants, our study confirms that the situation becomes more complicated. Hence, it may not be possible to assign correct potency values to a recombinant streptokinase product using the 3rd international standard for streptokinase and the chromogenic method now adopted by the European and British Pharmacopeias. Indeed, according to WHO guidelines, each recombinant biological and mutant variant being developed for therapeutic use should be evaluated with their own standard (20). Therefore, unless an appropriate international standard for recombinant streptokinase is developed, the chromogenic assay method described in EP and BP may not give an accurate indication of the potency of recombinant streptokinase. 
